# Mapping stillbirth and infant mortality rates: a district-wise exploration using civil registration system reports of Karnataka

**DOI:** 10.1097/j.pbj.0000000000000294

**Published:** 2025-06-09

**Authors:** Deepthy Melepurakkal Sadanandan, Kalesh M. Karun, Ashwini Dandappanavar, Umesh S. Charantimath, Manjunath S. Somannavar, Shivaprasad S. Goudar

**Affiliations:** a Women’s & Children’s Health Research Unit, Jawaharlal Nehru Medical College, KLE Academy of Higher Education and Research, Belagavi, Karnataka, India; b ICMR—National Institute of Traditional Medicine, Belagavi, Karnataka, India

**Keywords:** infant mortality rate, Karnataka, spatial analysis, stillbirth rate

## Abstract

**Background:**

Reducing stillbirth and infant mortality rates has become the primary focus of global public health initiatives to improve maternal and child health. This study aimed to investigate the temporal and spatial variations of stillbirth and infant mortality in Karnataka using the Civil Registration System (CRS) reports, examining trends from 1971 to 2022.

**Methods:**

District-wise summaries of stillbirth and infant mortality rates from CRS reports were analyzed using geospatial techniques, including choropleth mapping and spatial autocorrelation, to assess their distribution across Karnataka districts for selected years (2001, 2010, and 2022).

**Results:**

The findings reveal distinct temporal patterns in stillbirth rates over the study period, allowing for the identification of areas of concern. Dharwad district exhibited the highest stillbirth rate (16.67 per 1,000 total births), whereas Chamarajanagara reported highest infant mortality rate (21.88 infant deaths per 1,000 live births) in 2022. As per 2022 CRS reports, both stillbirth and infant mortality rates were highest in Dharwad, Gadag, Davangere, and Chitradurga districts. Spatial analysis techniques highlight clusters and disparities in stillbirth occurrences among different districts, providing valuable insights into regional variations.

**Conclusions:**

This study highlights a declining trend in the number of stillbirths and reveals substantial spatial disparities in both stillbirth and infant mortality rates across Karnataka. The identification of high-burden districts such as Dharwad, Gadag, Davangere, and Chitradurga emphasizes the need for geographically targeted strategies to improve maternal and child health outcomes.

## Abstract 2

Supplemental Digital Content is Available in the Text.

## Introduction

Stillbirth, the loss of a fetus at or after 28 weeks of gestation before birth, is a major global public health issue.^1^ It not only has profound emotional and psychological impacts on families but also reflects the overall maternal and fetal health within a population. Consequently, reducing stillbirth and infant mortality rates has become the primary focus of public health initiatives globally for improving maternal and child health.

The global stillbirth rate (SBR) was estimated to be 13.9 stillbirths per 1,000 total births in 2019.^2^ As per the National family health survey (2016–2021), India's SBR was 9.7/1000, with significant variation across states.^3^ Tripura reported the highest rate (13.9/1000), while Kerala had the lowest (1.5/1000) and Karnataka reported 6.3/1000. Belgaum experienced SBR of 26/1000 and 24 neonatal deaths per 1000 live births between 2014 and 2017 as reported in the study conducted by Dhaded et al.^4^ As per the fifth National Family Health Survey (NFHS), the overall estimated neonatal mortality rate in India was 24.8/1000 during 2019–2021.^5^ Despite continuous reductions in infant and younger than 5 years child mortality in India, these improvements remain unevenly distributed across various states and population groups.^6^

Infant mortality rate (IMR) is an indicator of health status of a community and effectiveness of maternal health services. Thus, tracking SBR and IMR is critical for identifying areas for improvement and implementing preventive measures. This study aims to map the district-level variations in SBR and IMR of Karnataka using district specific data with the help of geospatial technology. Comparison of district-wise distribution of SBR and IMR for the years 2001, 2010 and 2022 was also done.

## Methods

### Brief description about data

This study was based on secondary data extracted from Civil Registration System (CRS) reports of Karnataka state from 1971 to 2022.^7^ The CRS in India serves as a vital mechanism for recording and documenting vital events such as births and deaths. It provides a foundational source of data that is crucial for demographic analysis, public health planning, and policy formulation. The information on stillbirth was procured from CRS reports from 1971 to 2022. For different years, district-wise specific data on urban, rural, and overall stillbirth and infant mortality rates were extracted for 2001, 2010, and 2022 and are provided in the supplementary file, http://links.lww.com/PBJ/A44. Karnataka currently comprises 31 districts. Up to 2020, Vijayanagara was a part of Bellary district, and later in 2021, it was carved out of Bellary as the 31st district of Karnataka state.^8^ Thus, the CRS reports of Karnataka for the year 2022 consist of 31 districts. However, as the shapefile of Karnataka does not include Vijayanagara, it was considered as a part of Bellary for analysis purpose. Ethical approval was not required as the research solely relied on secondary data sourced from CRS reports.

### Statistical analyses

Stillbirth rate is defined as the number of stillbirths divided by the sum of stillbirths and live births. Trend in the number of stillbirths was plotted for the period from 1971 to 2022.Geospatial maps (Choropleth maps) were created to visualize the distribution of stillbirths and infant mortality rates across different districts in Karnataka and identify high-risk and low risk areas. Different color intensities on the Choropleth map indicated the variation in the magnitude of stillbirths. Moran's I statistic was used to estimate spatial autocorrelation in stillbirth rates and infant mortality rates.^9^ Moran's I is a measure used in spatial statistics to assess the degree of spatial autocorrelation in a set of geographic data. It helps in identifying clustering or dispersion patterns in spatial data. If the Moran's I is −1 or +1 indicate perfect negative or positive spatial autocorrelation, whereas 0 indicate random spatial pattern. All the statistical analyses were performed using R software version 4.2.3. A *P*-value less than 0.05 was considered as statistically significant.

## Results

### Trend in the number of stillbirths

**Figure 1.**
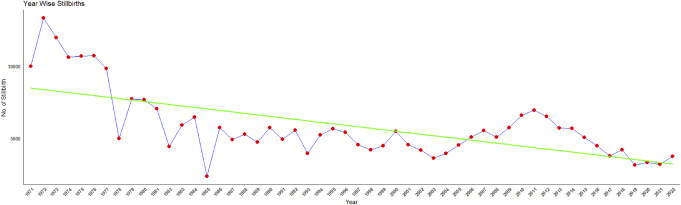
Trend in the number of stillbirths reported in Karnataka (CRS reports: 1971–2022).

Overall, the number of stillbirths in Karnataka showed a declining trend from 1971 to 2022, as illustrated in Figure 1. The highest was reported in the year 1972 (26.7/1000, n = 13,312) and least in 2019 (3.0/1000, n = 3156).

### Spatial distribution of stillbirth rates

**Figure 2.**
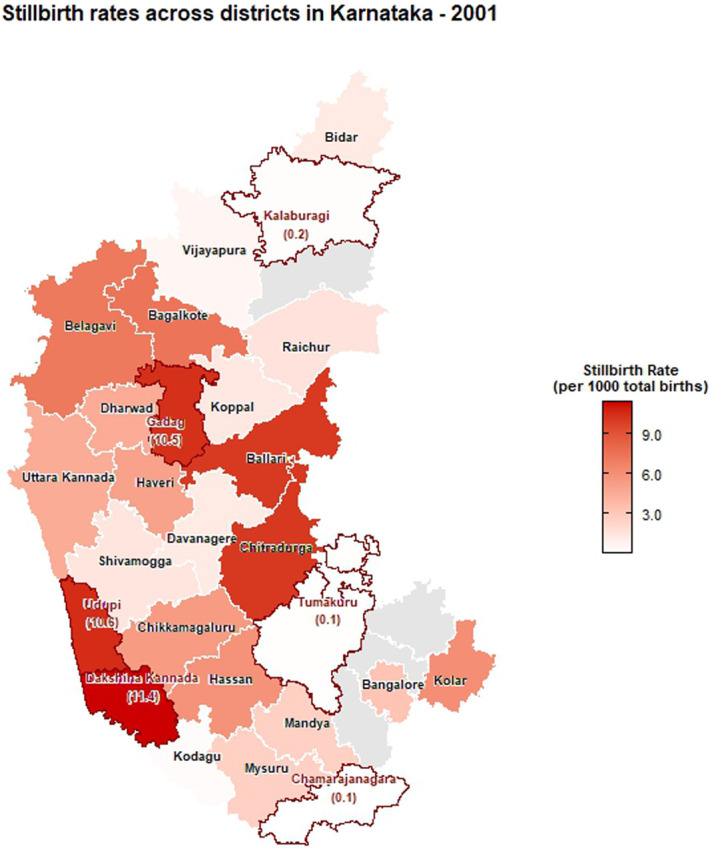
Choropleth map showing district-wise stillbirth rates in Karnataka for the year 2001.

**Figure 3.**
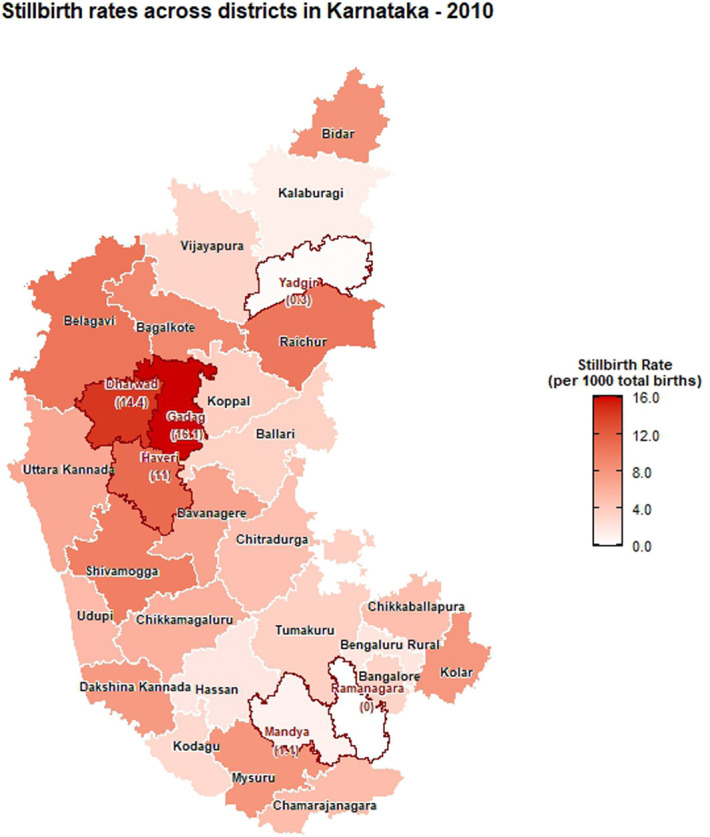
Choropleth map showing district-wise stillbirth rates in Karnataka for the year 2010.

**Figure 4.**
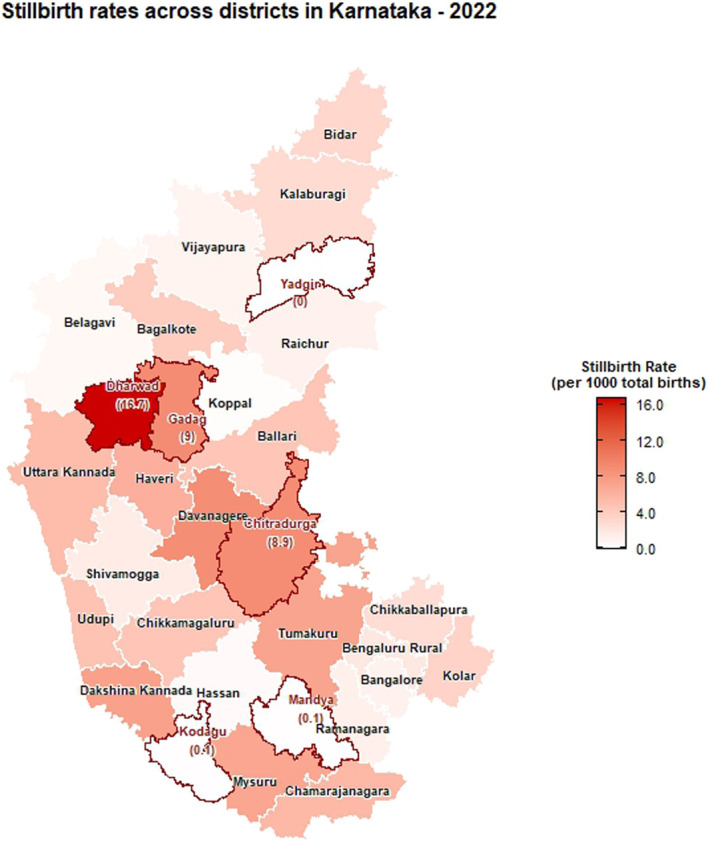
Choropleth map showing district-wise stillbirth rates in Karnataka for the year 2022.

**Figure 5.**
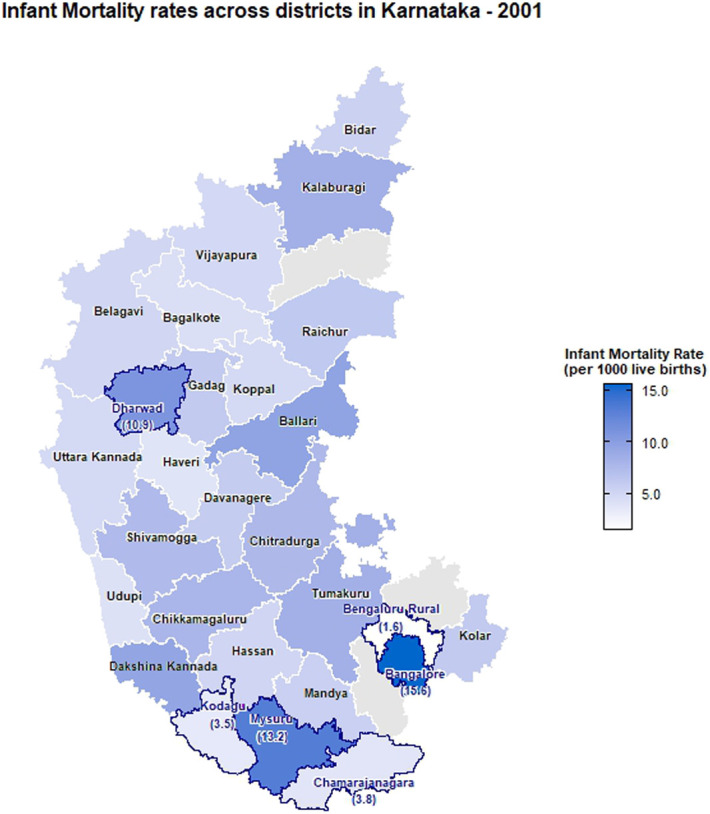
Choropleth map showing district-wise infant mortality rates in Karnataka for the year 2001.

**Figure 6.**
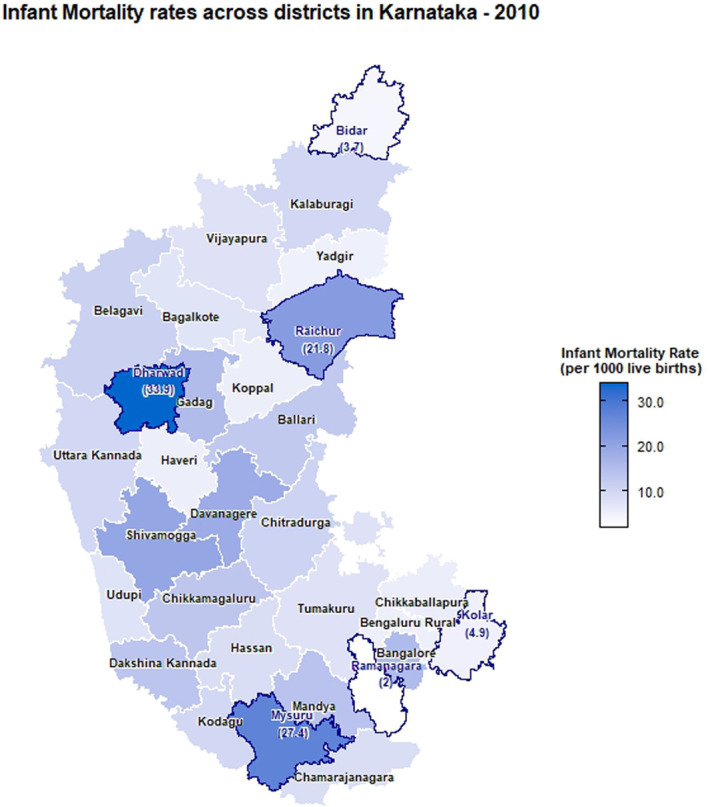
Choropleth map showing district-wise infant mortality rates in Karnataka for the year 2010.

**Figure 7.**
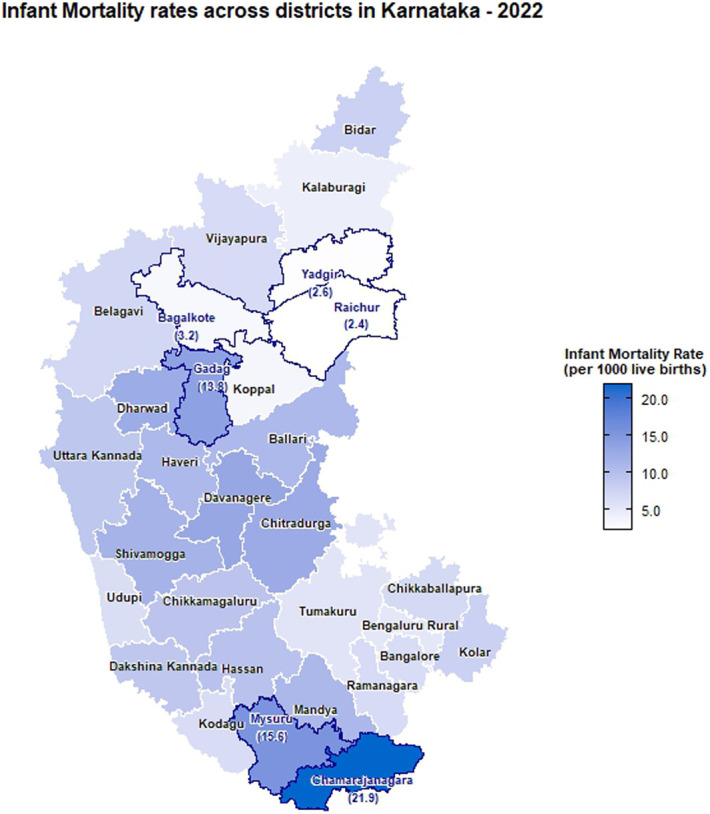
Choropleth map showing district-wise infant mortality rates in Karnataka for the year 2022.

The choropleth maps showing the spatial distribution of stillbirth and infant mortality rates across the 30 districts of Karnataka for the years 2001, 2010, and 2022 are presented in Figures 2–7. It provides information about the geographic perspective on the variations in stillbirth rates across the different districts of Karnataka. The variation magnitude of the stillbirth rate is denoted by increased intensity of colors where the gradation from light to dark shades signifies the magnitude of the event. Darker colors on the map correspond to higher stillbirth rates, highlighting districts with increased occurrences.

Great geographical disparity in the stillbirth rates was observed across different districts of Karnataka in 2001. With 11.42 stillbirths per 1000 total births, Dakshina Kannada exhibited highest SBR among Karnataka districts, followed by Udupi (10.6), Gadag (10.47), Chitradurga (10.16), and Ballari (10.09), with the lowest rate in Chamarajanagara district (0.06). By 2010, Gadag had the highest SBR at 16.08, followed by Dharwad (14.36), Haveri (11.03), Belagavi (10.37), and Raichur (10.23), with Yadgir (0.30) and Ramanagara (0) having the lowest rates. In 2022, Dharwad recorded the highest SBR among Karnataka districts at 16.67, followed by Gadag (9.01), Chitradurga (8.94), and Davanagere (8.88). The lowest rates were noted in Kodagu (0.13) and Yadgir (0). As per 2022 CRS report, the districts in the middle part of Karnataka exhibited high stillbirth rate when compared with its northern and southern end. In addition, among the aspirational districts, the highest stillbirth rates were reported as 9.01 in Gadag, followed by 2.81 in Gulburga, 1.11 in Raichur, and 0 in Yadgir. Significant reductions in SBR were observed in Belagavi, Raichur, and Shivamogga from 2010 to 2022. In addition, a moderate reduction was observed in the SBR for Gadag district in 2022 when compared with 2010. Dharwad has consistently had a high SBR across the years, indicating persistent issues in maternal and infant health care.

Among the urban areas, the highest stillbirth rate was observed in Udupi (36.73), Bagalkote (12.56), and Chitradurga (46.27) in 2001, 2010, and 2022, respectively (Supplementary file, http://links.lww.com/PBJ/A44), whereas among rural areas, Udupi (5.76), Dharwad (22.18), and Uttara Kannada (0.61) had the highest SBR in 2001, 2010, and 2022, respectively.

### Spatial distribution of infant mortality rates

In 2001, the highest IMR was observed in Bangalore (15.56 infant deaths per 1,000 live births), followed by Mysuru (13.22) and Dharwad (10.94) with the least being reported at Bengaluru rural (1.56). As per the 2010 CRS report, Dharwad (33.9) ranked highest, followed by Mysuru (27.36), Raichur (21.84), Shivamogga (19.73), and Ramanagara (2.04) with the least. In the year of 2022, the highest infant mortality rate was reported in Chamarajanagara (21.88), followed by Mysuru (15.59) and Gadag (13.8), and the least was reported in Raichur (2.37). Intensity of infant mortality was again high in the middle part and southern end when compared with the northern pole of Karnataka in 2022.

There are notable fluctuations in some districts, especially Raichur showed a significant decrease from 21.84 in 2010 to 2.37 in 2022, indicating a dramatic improvement. Significant improvements are also seen in districts such as Dharwad and Mysuru, which had very high IMRs in 2010 but showed substantial reductions by 2022. Districts such as Koppal and Yadgir have consistently maintained low IMRs across the years, indicating better healthcare and infant care practices. Some districts such as Chamarajanagara showed an increase in IMR, rising from 8.47 in 2010 to 21.88 in 2022, indicating a worsening situation. Both stillbirth and infant mortality rates were higher in Dharwad, Gadag, Davangere, and Chitradurga districts.

Urban regions of Chamarajanagara, Chitradurga, and Mysuru showed higher IMR as per 2022 CRS reports, whereas Bagalkote reported the lowest IMR. Raichur showed a significant decrease in IMR from 31.62 to 4.54 from 2010 to 2022 in the urban regions. However, Chamarajanagara district raised the concerns in 2022 with a jump in the IMR from 12.38 in 2010 to 29.15. Rural regions of Chamarajanagara, Mysuru, and Dharwad also exhibited highest IMR in 2022. However, significant improvement was observed in the IMR among the rural regions of Dharwad district, decreasing from 59.93 in 2010 to 13.43 in 2022.

### Spatial autocorrelation

The Moran's I test statistic was computed to assess spatial autocorrelation in stillbirth rates and infant mortality rates. Moran's I measures how similar or dissimilar spatial data points are to nearby data points. For stillbirth rates, the Moran's I statistic was 0.16 suggesting a significant positive spatial autocorrelation indicating clustering of stillbirths in certain geographic areas, *P*-value = .049. The areas with high stillbirth rates tend to be located near other areas with high stillbirth rates, and the observed pattern is unlikely to occur by random chance. The Moran's I statistic for infant mortality rates, however, was −0.25 and was not statistically significant (*P*-value = .97), indicating no significant spatial autocorrelation. This suggests that the spatial distribution of infant mortality rates across the study areas is not distinguishable from a random spatial pattern. Overall, the results highlight the importance of considering spatial relationships when analyzing stillbirth rates, whereas infant mortality rates do not exhibit a spatial clustering pattern beyond what would be expected by chance.

## Discussion

The findings presented in this study shed light on the temporal trends and spatial distribution of stillbirth and infant mortality rates in Karnataka for the years 2001, 2010, and 2022. A declining trend in the number of stillbirths was observed over years from 1971 to 2022 in the state. Globally, also stillbirth rates showed a declining pattern from 2000 to 2019 (i.e., 21.4 to 13.9 stillbirths per 1000 total births).^2^ In 2018, the registered births and deaths in Karnataka revealed a continuous increase with minor fluctuations from 1971 to 2011, and the stillbirth rate witnessed a notable decrease during the same period indicating positive development.^10^ The observed overall decline in stillbirth rates is encouraging, indicative of advancements in health care, prenatal care, and public health initiatives over the decades. However, the detailed spatial analysis reveals considerable heterogeneity across the 30 districts of Karnataka, necessitating a targeted approach to address specific challenges.

The choropleth maps presented offer a visual representation of the spatial patterns of stillbirth and infant mortality rates, providing valuable insights into regional disparities. The color-coded spatial maps serve as an effective visual tool for identifying and understanding the spatial distribution and relative intensity of stillbirth rates across the diverse districts of Karnataka in different years. Different colors on the maps indicated variations in the magnitude of stillbirth rates, aiding in the identification of high-risk and low-risk areas. In 2022, among the 30 districts of Karnataka, Dharwad exhibited the highest stillbirth per 1000 total birth (16.67). The prevalence of high-risk pregnancies was determined to be 37% in a cross-sectional study conducted in Dharwad in 2015, which also raised concerns.^11^ The identification of hotspots such as Dharwad, Gadag, Chitradurga, and Davangere with higher stillbirth rates emphasizes the urgency of tailored interventions in these areas. These districts demand focused attention for resource allocation, implementation of awareness programs, and improvement of healthcare infrastructure to address the root causes contributing to elevated stillbirth rates. Hypertensive disorders, intrapartum fetal loss, and placental abruption are few identified primary causes of stillbirth.^12^ In addition, factors such as nutritional anemia, teenage pregnancy, and intrauterine growth restriction have also been associated with an increased risk of stillbirth. Improved maternal nutrition interventions, accessible and quality obstetric services, timely management of hypertension, and complications during labor can help prevent stillbirth to a certain extent.^13^

A study conducted in the rural regions of 8 districts in Northern Karnataka in 2015 reported an infant mortality rate of 44 deaths per 1000 live births and a neonatal mortality rate of 33.5 deaths per 1000 live births.^14^ As per the CRS report of 2022, in Karnataka, the highest infant mortality rate was reported in Chamarajanagara (21.88). Furthermore, districts such as Mysuru, Gadag, and Davanagere with high infant mortality rates are areas with varying degrees of concern. The identification of these areas with high infant mortality rates reinforces the need for comprehensive healthcare strategies, including maternal and neonatal care, vaccination programs, and community education. It is also essential to implement awareness programs in these communities, focusing on educating expectant mothers and families about prenatal care, vaccination, and maternal and neonatal health.

The intertwining nature of stillbirth and infant mortality rates in specific regions, particularly Dharwad, Gadag, Davangere, and Chitradurga, emphasizes the interconnected challenges faced by these regions. A geospatial analysis conducted using NFHS data revealed that regions that suffered from inadequate child nutrition, limited wealth, or low female literacy were also likely to have lower rates of infant and child survival.^15^

Collaborative efforts are essential to address these challenges comprehensively. This includes the involvement of healthcare authorities, government agencies, nongovernmental organizations, and local communities working together to implement sustainable interventions. Future translational and implementation research should delve into the immediate and distal risk factors contributing to stillbirth and infant mortality rates in the identified hotspots, incorporating qualitative insights from both the healthcare system and the community at large.^16^ This approach will facilitate targeted and evidence-based interventions to address the underlying causes effectively.

## Conclusions

The identification of hotspots such as Dharwad, Gadag, Chitradurga, and Davangere with higher stillbirth rates and infant mortality rates emphasizes the urgency of tailored interventions in these areas. The article provides a baseline for ongoing monitoring and evaluation, essential for assessing the effectiveness of implemented interventions over time. In conclusion, this study contributes to the understanding of stillbirth and infant mortality trends across districts in Karnataka, providing a foundation for evidence-based policy decisions and targeted public health initiatives.
